# Outcomes of Salvage Robotic-assisted Radical Prostatectomy in the last decade: systematic review and perspectives of referral centers.

**DOI:** 10.1590/S1677-5538.IBJU.2023.0467

**Published:** 2024-02-07

**Authors:** Marcio Covas Moschovas, Carlo Andrea Bravi, Paolo Dell'Oglio, Filippo Turri, Ruben de Groote, Nikolaos Liakos, Mike Wenzel, Christoph Würnschimmel, Fabrizio Di Maida, Federico Piramide, Iulia Andras, Alberto Breda, Alexandre Mottrie, Vipul Patel, Alessandro Larcher

**Affiliations:** 1 AdventHealth Global Robotics Institute Florida USA AdventHealth Global Robotics Institute, Florida, USA; 2 University of Central Florida Florida USA University of Central Florida (UCF), Florida, USA; 3 ORSI Academy Ghent Belgium ORSI Academy, Ghent, Belgium; 4 The Royal Marsden NHS Foundation Trust Department of Urology London UK Department of Urology, The Royal Marsden NHS Foundation Trust, London, UK; 5 ASST Grande Ospedale Metropolitano Niguarda Milan Italy ASST Grande Ospedale Metropolitano Niguarda, Milan, Italy; 6 La Statale University ASST Santi Paolo e Carlo Milan Italy ASST Santi Paolo e Carlo - La Statale University, Milan, Italy; 7 OLV Hospital Department of Urology Aalst Belgium Department of Urology, OLV Hospital, Aalst, Belgium; 8 University of Freiburg Medical Centre Germany University of Freiburg Medical Centre, Germany; 9 University Hospital Frankfurt Germany University Hospital Frankfurt, Germany;; 10 Luzerner Kantonsspital Switzerland Luzerner Kantonsspital, Switzerland;; 11 University of Florence Florence Italy University of Florence, Florence, Italy;; 12 University of Turin San Luigi Gonzaga Hospital Italy University of Turin, San Luigi Gonzaga Hospital, Italy; 13 Iuliu Hatieganu University of Medicine and Pharmacy Cluj-Napoca Romania Iuliu Hatieganu University of Medicine and Pharmacy, Cluj-Napoca, Romania;; 14 Autonoma University of Barcelona at Fundacio Puigvert Barcelona Spain Autonoma University of Barcelona at Fundacio Puigvert, Barcelona, Spain;; 15 San Raffaele Hospital Milan Italy San Raffaele Hospital, Milan, Italy

**Keywords:** Salvage Robotic-assisted radical prostatectomy, prostate cancer recurrence, robotic surgery

## Abstract

**Purpose:**

Salvage robotic-assisted radical prostatectomy (S-RARP) has gained prominence in recent years for treating patients with cancer recurrence following non-surgical treatments of Prostate Cancer. We conducted a systematic literature review to evaluate the role and outcomes of S-RARP over the past decade.

**Materials and Methods:**

A systematic review was conducted, encompassing articles published between January 1st, 2013, and June 1st, 2023, on S-RARP outcomes. Articles were screened according to PRISMA guidelines, resulting in 33 selected studies. Data were extracted, including patient demographics, operative times, complications, functional outcomes, and oncological outcomes.

**Results:**

Among 1,630 patients from 33 studies, radiotherapy was the most common primary treatment (42%). Operative times ranged from 110 to 303 minutes, with estimated blood loss between 50 to 745 mL. Intraoperative complications occurred in 0 to 9% of cases, while postoperative complications ranged from 0 to 90% (Clavien 1-5). Continence rates varied (from 0 to 100%), and potency rates ranged from 0 to 66.7%. Positive surgical margins were reported up to 65.6%, and biochemical recurrence ranged from 0 to 57%.

**Conclusion:**

Salvage robotic-assisted radical prostatectomy in patients with cancer recurrence after previous prostate cancer treatment is safe and feasible. The literature is based on retrospective studies with inherent limitations describing low rates of intraoperative complications and small blood loss. However, potency and continence rates are largely reduced compared to the primary RARP series, despite the type of the primary treatment. Better-designed studies to assess the long-term outcomes and individually specify each primary therapy impact on the salvage treatment are still needed. Future articles should be more specific and provide more details regarding the previous therapies and S-RARP surgical techniques.

## INTRODUCTION

Since the first robotic platform was cleared by the FDA (Food and Drug Administration) in 2000, robotic surgery has expanded and improved in multiple fields with the use of different consoles in urologic surgeries. Currently, radical prostatectomy (RP) is the most common urologic procedure performed with robotic assistance, and it is the gold-standard surgical treatment for localized prostate cancer (PCa) in centers with access to robotic surgery (1). However, despite these technological advancements, other less-invasive therapies and technologies have also been described, and the surgical approach is not the only option available for treating PCa.

Concurrent with robotic surgery development, the armamentarium for treating prostate cancer without surgery is vast in the current literature, including several modalities of radiation therapy (RT) (2, 3) and different techniques of focal therapy (FT) (4, 5). However, the best management for the local cancer recurrence after a non-surgical primary treatment is still under discussion, and despite the variety of non-surgical treatments for localized PCa, every therapy causes anatomical modifications and consequences that will impact the outcomes of a subsequent salvage robotic-assisted radical prostatectomy (S-RARP).

As a minimally invasive surgical approach, due to increased experience by robotic surgeons, S-RARP has rapidly gained momentum over the last decade, transforming the landscape of prostate cancer salvage therapy. Unlike traditional open surgeries, robotic-assisted techniques employ advanced technology, enabling surgeons to achieve unparalleled dexterity and visual magnification. This revolution in surgical technology has allowed for greater preservation of critical anatomical structures, leading to reduced rates of postoperative complications and improved functional outcomes. However, even with robotic surgery advantages, S-RARP is still a challenging procedure for surgeons and patients. Therefore, we performed a systematic literature review assessing the outcomes and the robotic surgery role in the past decade to approach salvage radical prostatectomy.

## EVIDENCE ACQUISITION

### Literature Search Strategy

We performed a systematic literature review (PROSPERO number CRD42023429052) of articles published in the last ten years (from January 1st, 2013 to June 1st, 2023), assessing the available studies describing outcomes of salvage robotic-assisted radical prostatectomy (S-RARP). The literature screening included PubMed^®^, Web of Science, and Cochrane using the terms "salvage robotic-assisted radical prostatectomy, salvage robotic laparoscopic prostatectomy, and salvage robotic radical prostatectomy."

Afterward, two investigators (M.C.M and C.B) independently screened and checked all articles using standardized data extraction forms. In case of any discrepancy about eligibility, the article was evaluated by a third author (P.D). The review was performed according to preferred reporting items for Systematic reviews and Meta-analyses (PRISMA) guidelines (6, 7).

### Inclusion and exclusion criteria

**Figure 1:**
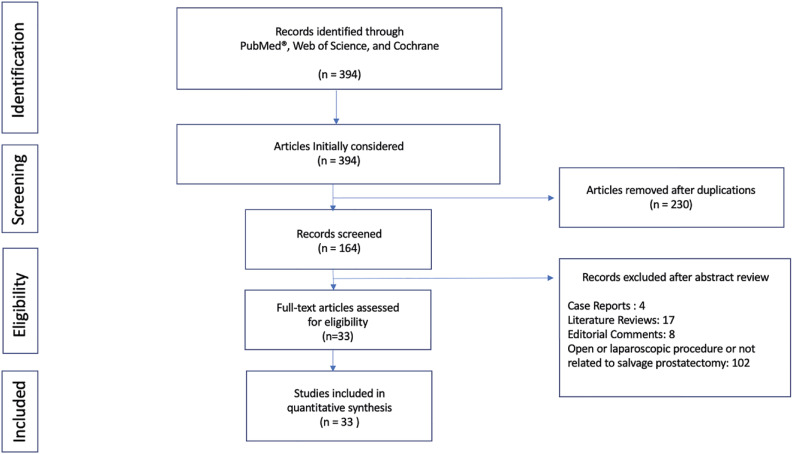
Flowchart illustrating the literature search with exclusion criteria until reaching the number of articles used in the review.

We selected articles written in English describing research and studies in humans. When finding studies from the same institution with overlapping patients and outcomes, we considered only the most recent data. We also excluded previous literature reviews, studies mixing the data of different approaches (open, laparoscopic, and robotic) without specifying the robotic surgery outcome, conference abstracts, and case reports of techniques previously described in the literature. When evaluating studies with different groups of S-RARP, we considered only the robotic surgery data. Figure-1 illustrates a flowchart with the selection criteria used. References were manually reviewed and reported according to the PubMed^®^ citation format.

### Variables and outcomes definition

We included relevant data regarding the year of the article publication: number of patients undergoing S-RARP in the study, type of primary therapy, total operative time (TOT), console time (CT), intraoperative complications (IOC), estimated blood loss (EBL), postoperative complications (POC), positive surgical margins (PSM), continence recovery rates, potency recovery rates, biochemical recurrence (BCR), and median follow-up (MFU).

The primary endpoint of our review is to evaluate and report complication rates, functional and oncological outcomes of S-RARP.

## EVIDENCE SYNTHESIS

### Summary of the studies

Similar to the primary intervention, the salvage treatment for prostate cancer can be performed with different approaches, such as surgery, RT, HIFU, Cryo, EBRT, and androgen deprivation. However, in this section, we will describe the literature specific on the surgical treatment with salvage robotic-assisted radical prostatectomy (S-RARP).

Overall, we identified 394 articles that fulfilled our search criteria, of which 230 were duplications. From 164 remaining articles, we excluded 131 after the abstract review. In summary, 33 articles were selected containing the robotic surgery approach to patients who underwent previous non-surgical treatment for prostate cancer (Figure-1) (8-40).

Overall, we identified a total of 33 articles with a median follow-up that ranged from 7 to 44 months. Table-1 illustrates the articles from 2013 to 2019, while Table-2 illustrates articles from 2020 to 2023. The tables include the data collected on each study, including the year of publication, the number of patients of each study, type of primary therapy, total operative time (TOT), console time (CT), intraoperative complications (IOC), estimated blood loss (EBL), postoperative complications (POC) according to Clavien-Dindo (Clv), positive surgical margins (PSM), continence, potency, biochemical recurrence (BCR), and median follow-up (MFU) in months.

### Primary therapy for PCa before S-RARP

Overall, in the past 10 years, a total of 1630 patients reported in the literature underwent S-RARP, with radiotherapy being the most common primary treatment (42%), followed by HIFU (18%), brachytherapy (10%), and cryotherapy (5%). The remaining patients (25%) underwent other therapies or a combination of therapies as primary treatment for PCa.

### Surgical Performance and Complications

The total operative time range was from 110 to 303 minutes (reported by most articles), and the console time ranged from 84 to 199 minutes (reported by few studies).(12,20,23,36) Estimated blood loss ranged from 50 to 745 mL.(23,30) Intra- and postoperative complications ranged from 0 to 9,1% and 0 to 90%, respectively (Clavien 1 to 5). Rectal injury ranged from 0.48 to 3%. (8, 19, 25, 37).

### Functional Outcomes

Functional outcomes are illustrated in Tables 1 and 2. The continence rates ranged from 0 to 100% (14, 24, 33, 34), and the studies diverge regarding the definition of continence. Twenty-one studies defined continence as no pads use (10, 12, 15-22, 25, 26, 30, 31, 33, 35-40), five studies considered up to one pad (8, 9, 23, 28, 32), one study evaluated continence using EPIC-26 questionnaire 24, and one study considered the ICIQ-score questionnaire (29). We were not able to find continence definition in five studies (11, 13, 14, 27, 34).

**Table 1:** Articles included from 2013 to 2019.

Author	Year	N. of patients	Primary Therapy	TOT/CT (minutes)	IOC (%)	EBL (mL)	POC in 90 days (%) Clavien range	PSM (%)	Continence/Potency (%)	BCR (%)	MFU months
Kaffenberger et al. (8)	2013	34	BRC-19 RT-11 HIFU-4	NA/ NA	3 rectal	NA	30 (Clv 1-3)	26	39/21	18	16
Zugor et al. (9)	2014	13	EBRT-7 BRC-6	154/ NA	0	130	30 (Clv 1-3)	0	54/23	23	22.8
Yuh et al. (10)	2014	51	BRC-23 Cryo- 3 EBRT- 18 HIFU-1 PB-6	179/ NA	5	175	47 (Clv1-5)	31	45/23	43	36
Vora et al. (11)	2015	6	RT	NA/ NA	0	NA	16.7	NA	83.4/ NA	16.7	7.2
Kenney et al. (12)	2016	20	RT	303/ NA	5	381	30 Clv ≥3	15	NA/ NA	22	9.5
Ozu et al. (13)	2016	1	HIRT	244/ 189	0	100	0	0	NA/NA	0	10
Peretsman et al. (14)	2017	9	HIFU (Sonablate)	130/ NA	0	125	NA	NA	100/20	NA	NA
Orré et al. (15)	2017	7	BCR	148/ NA	0	NA	14 Clv ≥3	14	50/ 66	14	24
Ou et al. (16)	2017	14	RT-11 CKR-2 HIFU-1	NA/ 134	7	100	21	21.4	71.4/ 66.7	21	32.4
Nunes-Silva et al. (17)	2017	22	FT	134/ NA	9.1	465	NA	4.5	53.8/ NA	31.8	12
Ogaya-Pinies et al. (38)	2018	60	EBRT-35 BCR-10 PB-6 Cryo-7 HIFU-2	131/ NA	0	130	5 Clv 1-2	NA	50 / NA	NA	12
Bonet et al. (22)	2018	80	RT-63 ABL-17	NA/ NA	0	NA	NA	NA	53.7/ 16.6	31	22.5
Ogaya-Pinies et al. (40)	2018	96	EBRT-37 BCR-14 BCR+EBRT-13 CYK-3 PB-1 Cryo-18 HIFU-7 Others 4	125/ NA	0	100	26 Clv 1-4	16.7	57.3/ 55	15	14
Marconi et al. (18)	2019	82	HIFU-57 Cryo-16 IRE-4 VTP-3 PRX302-2	NA/ NA	0	400	6.1 Clv 1-3b	13.4	83/ 14	41.5	13
Gontero et al. (19)	2019	209	RT-121 BCR-55 Cryo-14 HIFU-9 Others-10	228/ NA	0.48 rectal	222	34.9 Clv1-4	NA	63.9/ 8.1	NA	28.8
Onol et al. (20)	2019	126	RT-94 ABL-32	129/84 122/84	0 0	106 93	20 Clv 1-4 9 Clv 1-3a	17 43.8	51.3/ 13 87.5/ 27	17 18	32 29

Clv (Clavien-Dindo); EBL (estimated blood loss); PSM (positive surgical margins); TOT (total operative time); CT (console time); IOC (intraoperative complication); POC (postoperative complications); NA (not available); MFU (median follow up); PB (proton bean); Cryo (cryotherapy); CYK (Cyberknife); BCR (biochemical recurrence on the period of the study); Primary Therapy: RT (radiotherapy); HIRT (heavy iron radiotherapy therapy); BRC (Brachytherapy); HIFU (high intensity focused ultrasound); FT (focal therapy); EBRT (external-bean radiotherapy); ABL (ablation); IRE (Irreversible Electroporation) VTP (vascular-targeted photodynamic therapy); PRX302 (Topsalysin)

**Table 2:** Articles included from 2020 to 2023.

Author	Year	N. of patients	Primary Therapy	TOT/CT (minutes)	IOC (%)	EBL (mL)	POC (Clavien) In 90 days (%)	PSM (%)	Continence/Potency (%)	BCR (%)	MFU months
Thompson et al. (21)	2020	45	HIFU	NA/ 140	0	200	17.8 Clv 1-3	44.4	65.5/ 0	5.3	17.7
Bonet et al. (39)	2020	120	RT ABL	127/ 84	0	103	14.2 Clv1-4	22.5	55,8/ 19.2	32.5	44
De Groote et al. (35)	2020	106	HIFU-59 RT-27 BRC-10 Others -10	142/NA	0	200	8 Clv 1-3	39	50/ 5	13	25
Madi et al. (23)	2021	*RS (20) Usual (6)	EBRT-18 BRC-4 Cryo-2 CYK-2	NA/ 141 NA/ 199	0 0	50 100	10 Clv 1-3 16,7 Clv 3	30 33	100/NA 44/NA	20 33,3	18
Cathcart et al. (24)	2021 (RCT)	23	Cryo (4) HIFU (17) Electro (1)	NA	0	NA	4 (Clv 1)	35	100/52	18	12
Martinez et al. (25)	2021	26	BRC (3) EBRT (19) IMRT (3)	NA	3.8 rectal	150	11.5(Clv 3-5)	26,9	90.9/4.5	39.1	47
Nathan et al. (26)	2021	135	N/A	165/NA	0.8 rectal	200	13.3 (Clv 1-5)	37.8	78.8/5.2	22.2	43
Nunes-Silva (27)	2021	*RS (12)	EBRT (10) BRC (2)	NA/138	0	81	8.3(Clv 3)	25	91.6/NA	16.6	12
Kowalczyk et al. (28)	2021	*RS (40)	EBRT (21) BRC (12) HIFU (7)	NA/130	2.5	100	12.5 (Clv 1-5)	57.5	54.1/10	23.1	23
Kowalczyk et al. (28)	2021	32	EBRT (16) BRC (9) Cryo (5) RT (2)	NA/175	0	150	28.1(Clv 1-3)	65.6	6.3/12.5	37.5	36
Spitznagel et al. (29)	2021	13	HIFU	260/NA	0	230	46.2 (Clv 1-3)	7.7	NA/NA	0	12
Bozkurt et al. (30)	2021	10	PTB	230/NA	0	745	90 (Clv 1-4)	20	12.5/0	10	31.8
Bhat et al. (31)	2021	53	FT	NA/121	0	100	21 (Clv1-2)	40	56/13	17	36.3
Schuetz et al. (32)	2021	*RS (21)	RT (8) HIFU (9) BRC (2) Cryo (1) FT (1)	228/NA	0	300	NA	19	19/NA	14.3	12
Schuetz et al. (32)	2021	7	RT (4) HIFU (1) BRC (2)	252/NA	0	500	NA	57,1	0/NA	57,1	36
Blazevski et al. (33)	2022	15	FT	NA/NA	0	200	NA	7	100/60	0	22
Mortensen et al. (34)	2022	5	EBRT	205/NA	0	120	60 (Clv 1-3)	60	0/NA	20	13
Nathan et al. (37)	2022	100	HIFU (92) Cryo (5) IRE (2)	170/NA	0	200	9 (Clv 1-3)	25	84,7/21	23	16,5
De Luca et al. (36)	2022	11	HIFU	110/NA	0	NA	20 (Clv 1)	27	81/18	0	12

Clv (Clavien-Dindo), EBL (estimated blood loss), PSM (positive surgical margins), TOT (total operative time), CT (console time), IOC (intraoperative complication), POC (postoperative complications), NA (not available), MFU (median follow up), PB (proton bean), Cryo (cryotherapy), CYK (Cyberknife), BCR (biochemical recurrence on the period of the study). Primary Therapy: RT (radiotherapy), HIRT (heavy iron radiotherapy therapy), BRC (Brachytherapy), HIFU (high intensity focused ultrasound), FT (focal therapy), EBRT (external-bean radiotherapy), IRE (Irreversible Electroporation), Electro (Electroporation), IMRT (Intensity-modulated radiotherapy),

* RS (Retzius-sparing), PTB (proton bean).

Potency rates ranged from 0 to 66.7% (16, 21, 30) and most studies defined potency as the capacity to have intercourse with or without phosphodiesterase 5 (PDE-5) inhibitors (8-10, 14-16, 18-22, 25, 26, 31, 33, 37, 39,40). One study evaluated potency recovery using the EPIC-26 questionnaire 24, and two studies using the IIEF-5 (29, 36). In some studies, we were not able to find data on potency (11-13, 17, 23, 27, 28, 30, 32, 34, 35, 38), continence, or BCR. Finally, most authors described the number of events in the follow-up period and failed to demonstrate functional outcomes in Kaplan-Meyer curves (time to event) to estimate early potency or continence following surgery.

### Pathological and Oncological Outcomes

Tables 1 and 2 describe the oncological outcomes. Positive surgical margins after S-RARP reached up to 65.6%(28), while biochemical recurrence ranged from 0 to 57% (32). All studies reported BCR according to RTOG-ASTRO Phoenix Criteria (1). However, in most studies, BCR in five years was not available due to the short-term follow-up. Most authors described BCR as the number of events in the follow-up period with percentages and failed to demonstrate it in Kaplan-Meyer curves (time to event) to estimate the recurrence time following surgery in a better fashion.

### Retzius-Sparing (RS) approach to S-RARP

Only four articles, comprising 93 patients, described the RS approach to S-RARP. In these studies, PSM ranged from 23.8 to 57.5% and continence from 19 to 100%. None of the studies described or compared early continence following RS. Potency was available in only one study (10%) (28). BCR ranged from 14.3 to 23.1% (23, 27, 28, 32).

## DISCUSSION

We have summarized the past decade of all studies describing outcomes of salvage robotic-assisted radical prostatectomy for prostate cancer recurrence after primary treatment. Reporting and comparing the outcomes of S-RARP is challenging because the available data is based on retrospective studies with a small number of patients and all its inherent risks of bias. The current literature is inconsistent, and most studies reported the overall outcomes adding different primary therapies without specifying or separating the patients and results according to the primary approach. Furthermore, the surgeries were performed by several surgeons with diverse levels of experience and techniques, which also impacts the outcomes of each patient (39). In addition, most studies failed to report the primary therapy details, such as radiation dose and fractions, type of energy used on the focal approach (full or partial ablation), side and histology of the primary tumor, and details of preoperative hormone treatment, which may impact in the positive surgical margins rates. In this scenario, due to the multifactorial risks of bias and inconsistencies, avoiding misleading conclusions regarding S-RARP, we reported and evaluated the current articles individually instead of conducting a meta-analysis.

Recent studies described the importance of selection criteria for S-RARP to optimize operative outcomes (41). According to the European Association of Urology (EAU), candidates for S-RARP should have low comorbidity, life expectancy of at least 10 years, PSA lower than 10, International Society of Urological Pathology (ISUP) grade group ≤ 2/3, no lymph node involvement on preoperative imaging exam, and clinical stage T1 or T2. Calleris and colleagues described significant differences and benefits for patients selected according to these variables. Unfortunately, being a recent study and guideline by EAU, most articles included in our review were published before 2022 and violated at least one criterion for selecting candidates for S-RARP (41). Therefore, we believe that outcomes of Robotic-assisted Salvage Prostatectomy should improve in the following years when selecting patients according to these guidelines.

Only one randomized clinical trial (RCT) was reported with a small number of patients (23), describing 100% continence rates after 12 months and 58% of potency recovery in patients treated with previous Focal Therapy (24). The largest series of S-RARP to date was published by Gontero et al., reporting the outcomes of 18 tertiary centers comparing open and robotic groups of salvage prostatectomy. In this retrospective study, 209 patients underwent S-RARP, and the author reported up to 34.9% of postoperative complication rates, with 0.48% of rectal injuries. Potency and continence for the robotic group reached 8.1% and 57.5%, respectively. PSM and BCR were not assessed in this study (19). Even with a relatively high number of patients reported by this study, evaluating functional and oncological outcomes in S-RARP is challenging due to several factors that impact surgical results. From this article, it is reasonable to conclude that intraoperative complications and rectal injury have low rates, but continence and potency recovery are suboptimal and challenging to perform a critical analysis of due to a lack of data on the preoperative function of these patients. In a subsequent study, the same group published a cohort of 414 patients describing the oncological outcomes. However, we excluded this data from our review because open and robotic results were mixed and not reported individually (42).

Some studies have assessed the S-RARP outcomes of patients selected according to the primary therapy. Two of them reported and compared the results of S-RARP following ablation and radiation. The authors had similar conclusions in terms of functional outcomes between the therapies (20, 35). In both articles, potency and continence rates were higher in patients who underwent primary ablations. However, the results are still suboptimal, even in the ablation group, which in theory had only partial damage on the prostate during the primary treatment. Kenney et al. also described the S-RARP in patients with previous radiation and reported complications with Clavien-Dindo ≥ 3 reaching up to 30%, while PSM and BCR reached 15% and 22%, respectively (12). In another study, Orré M et al. reported 14% of PSM and 14% of BCR in patients with previous brachytherapy (15). However, even with compatible results presented by these studies, PSM and BCR are also influenced by several factors such as tumor histology, previous hormone treatment, radiation fractions and dose, brachytherapy technique, and surgeon's experience.

Furthermore, nine articles including 374 patients exclusively reported the S-RARP following Focal Therapy (14, 17, 18, 21, 26, 29, 31, 33, 36). The complication rates ranged from 6.1 to 46.2%, PSM from 4.5% to 44%, and BCR from 0 to 41.5%. In these series, it is also challenging to stratify the patients due to a lack of information regarding the type of energy used in the Focal therapy (Focal One or Sonablate), whole or partial gland ablation, initial tumor histology and stage, preoperative potency, and continence. In this context, Bhat and colleagues described worse functional and oncological outcomes of S-RARP post Focal Therapy compared to primary radical prostatectomy, showing that focal ablations often cause contralateral prostate damage and also impact functional outcomes (31).

The Retzius-sparing approach to S-RARP has also been described in 4 studies comprising 93 patients (23, 27, 28, 32). The results were compatible with other series of S-RARP with intraoperative complications ranging from 0 to 2.5%, biochemical recurrence from 14.3 to 23%, continence from 19 to 100%, and most studies did not describe potency recovery rates. In one study, continence rates were 100% for the RS versus 44% for the conventional approach (27). However, due to the small number of cases reported and some missing data on outcomes in the literature, we still need more studies evaluating outcomes of this approach in salvage settings. The RS approach to S-RARP is feasible and safe when performed by experienced surgeons. We believe the challenge lies in the tumor recurrence and lack of landmarks posed by the primary treatment, not in the RS technique.

Among all possible intraoperative complications in patients undergoing S-RARP, rectal injury is one of the most feared by surgeons. Due to the technical challenges and lack of anatomic landmarks posed by the primary treatment, posterior adhesions between the prostate and rectum are common in this type of surgery. In addition, the primary therapy aggression on the rectum, especially radiation, usually makes the intestinal tissue more susceptible to dehiscence and fistula after an eventual repair. Evaluating the current articles in the literature, intraoperative complications ranged from 0 to 9%, and only three studies described rectal injury, being one patient with local staging pT4 disease (8,19,25). However, it is important to note that the literature on salvage prostatectomy is based on retrospective studies with great potential for selection bias, and we believe that the percentage of rectal injuries could be underestimated and underreported. In this scenario, patients and surgeons should be aware of the increasing risks of rectal injuries in these cases, and we recommend always having a general surgeon consultation and backup before operating on these patients.

Despite its strengths, our study is not devoid of limitations. First, as previously described, the current literature is based on retrospective studies and all its inherent limitations, especially selection bias. Second, most articles grouped different primary therapies and reported the outcomes without individualizing the primary approach with more details, such as radiation fractions and dose, previous hormone treatment and type of regimen (agonist or antagonist), type of ablation (whole or focal gland), and initial tumor histology and staging before primary treatment. Third, the articles have some inconsistencies in the definitions and reports of the trifecta (potency, continence, and BCR). Also, some studies reported only part of these outcomes. Fourth, most articles reported the overall rates of biochemical recurrence without specifying the time to recurrence on Kaplan-Meyer curves and the next steps on the treatment after recurrence following S-RARP. However, despite the literature limitations on S-RARP, we believe we could summarize the most important aspects of this challenging procedure, adding valuable inputs from experts in this field.

## CONCLUSIONS

In the last decade, salvage robotic-assisted radical prostatectomy in patients with cancer recurrence after previous prostate cancer treatment was safe and feasible. The literature is based on retrospective studies with inherent limitations describing low rates of intraoperative complications and small blood loss. However, potency and continence rates are largely reduced compared to the primary RARP series, despite the type of the primary treatment. In this scenario, patients should be aware and counseled about technical and anatomical challenges posed by the primary therapy and potential risk of rectal injury during the procedure. Better-designed studies to assess the long-term outcomes and individually specify each primary therapy impact on the salvage treatment are still needed. Future articles should be more specific and provide more details regarding the previous therapies and S-RARP surgical techniques.
